# Topological triple phase transition in non-Hermitian Floquet quasicrystals

**DOI:** 10.1038/s41586-021-04253-0

**Published:** 2022-01-19

**Authors:** Sebastian Weidemann, Mark Kremer, Stefano Longhi, Alexander Szameit

**Affiliations:** 1 Institute for Physics, University of Rostock, Rostock, Germany; 2 Dipartimento di Fisica, Politecnico di Milano, Milan, Italy; 3 IFISC (UIB-CSIC), Instituto de Fisica Interdisciplinar y Sistemas Complejos - Palma de Mallorca, Palma, Spain

**Keywords:** Micro-optics, Quantum simulation

## Abstract

Phase transitions connect different states of matter and are often concomitant with the spontaneous breaking of symmetries. An important category of phase transitions is mobility transitions, among which is the well known Anderson localization^1^, where increasing the randomness induces a metal–insulator transition. The introduction of topology in condensed-matter physics^2–4^ lead to the discovery of topological phase transitions and materials as topological insulators^5^. Phase transitions in the symmetry of non-Hermitian systems describe the transition to on-average conserved energy^6^ and new topological phases^7–9^. Bulk conductivity, topology and non-Hermitian symmetry breaking seemingly emerge from different physics and, thus, may appear as separable phenomena. However, in non-Hermitian quasicrystals, such transitions can be mutually interlinked by forming a triple phase transition^10^. Here we report the experimental observation of a triple phase transition, where changing a single parameter simultaneously gives rise to a localization (metal–insulator), a topological and parity–time symmetry-breaking (energy) phase transition. The physics is manifested in a temporally driven (Floquet) dissipative quasicrystal. We implement our ideas via photonic quantum walks in coupled optical fibre loops^11^. Our study highlights the intertwinement of topology, symmetry breaking and mobility phase transitions in non-Hermitian quasicrystalline synthetic matter. Our results may be applied in phase-change devices, in which the bulk and edge transport and the energy or particle exchange with the environment can be predicted and controlled.

## Abstract (web summary)

A triple phase transition, where changing a single parameter simultaneously gives rise to metal–insulator, topological and a parity–time symmetry-breaking phase transitions, is observed in non-Hermitian Floquet quasicrystals.

## Main

Phase transitions are defined as drastic changes of a system’s characteristics upon a small change of a single parameter. A classic example stems from chemistry, where changes between solid, liquid and gaseous phases can be induced by varying the temperature or pressure. In material sciences, the transition between the ferromagnetic and paramagnetic phases of magnetic materials at the Curie temperature is another fundamental example. The notion of phase transitions is established for understanding a diversity of different systems and phenomena, for instance, the evolution of planets in astrophysics^12^, intracellular functioning^13^ and the emergence of disease in biological systems^14^, Bose–Einstein condensation^15^, and the evolution of the early Universe and the formation of the fundamental forces^16–18^.

An important category among phase transitions—which are manifested in a plethora of different systems and phenomena—is mobility transitions^19,20^. These are a drastic change of the conductance, that is, the spreading and localization of quantum particles, upon altering a parameter beyond a critical point. When certain metals and ceramics are cooled below a critical temperature, superconductivity abruptly emerges^21^. Another seminal example is Anderson localization^1^, which is a sudden metal–insulator transition when uncorrelated randomness in a system is increased beyond a critical level.

A paradigmatic model showing a mobility transition is the Aubry–André–Harper (AAH) model^22^. It describes a one-dimensional system in an intermediate phase between perfect periodic order (crystal) and a completely disordered medium, possessing only long-range order without periodicity. This so-called quasicrystal^23^ is known for undergoing a metal–insulator phase transition in one dimension at a critical value of the potential strength^24,25^. At the critical point, the AAH model reduces to the Harper equation^26^ that can be directly mapped onto the two-dimensional Hofstadter model, which describes integer quantum Hall topology on a square lattice, resulting in the well known Hofstadter butterfly energy spectrum^27^.

Metal–insulator phase transitions have usually been regarded as unrelated to other types of phase transition, such as spontaneous symmetry breaking occurring in dissipative systems or topological phase transitions observed in topological matter. This common wisdom has been challenged by recent theoretical studies^9,10^, where the intriguing interplay between aperiodic order and dissipation has been unravelled. In such systems, the localization of the wavefunctions and the metal–insulator phase transition are usually associated with a spectral (symmetry breaking) phase transition^28^, which can be characterized by the change of a topological (winding) number emerging from the closed contours of the eigenvalue spectrum in the complex plane^9^. In general, non-Hermitian contributions, such as dissipation, break the Hermitian time-reversal symmetry. However, one might find a combined parity–time (PT) symmetry^6^, which allows for a completely real energy spectrum, if unbroken. The discovered exotic behaviour at the critical point of PT phase transitions has sparked numerous applications, such as enhanced sensing^29^, unidirectional invisibility^30^ and mode-selective vortex lasing^31^.

**Fig. 1:**
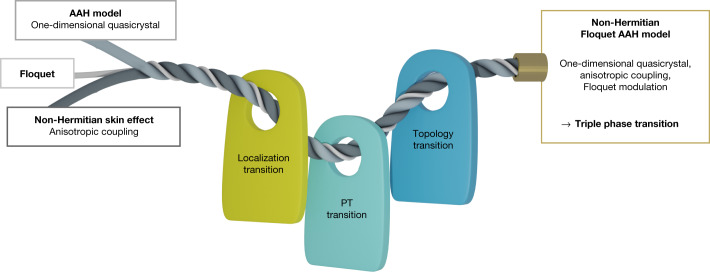
Intertwinement of the triple phase transition in the non-Hermitian Floquet AAH model. By intertwining a one-dimensional quasicrystal (AAH model) with a non-Hermitian anisotropy (skin effect model) via a temporal driving (Floquet mechanism), one obtains the non-Hermitian Floquet AAH model that is discussed in this work. Although none of the models on the left show a phase transition, except for a localization transition in the AAH model, the combined model on the right shows a triple phase transition, that is, a localization phase transition and a topological phase transition are connected to spontaneous PT symmetry breaking (energy-conservation transition). All three transitions occur at the same critical point of a single parameter, which can be a purely Hermitian parameter (the coupling strength) or a purely non-Hermitian parameter (the anisotropy strength).

Here we experimentally demonstrate a triple phase transition, where a topological phase transition, a mobility phase transition and spontaneous PT-symmetry breaking coincide (Fig. 1). We consider a non-Hermitian Floquet quasicrystal with PT symmetry, supporting the non-Hermitian skin effect^11,32,33^, and possessing non-trivial point-gap topology^9^. Remarkably, the triple phase transition is observed by changing a single parameter, which can be purely Hermitian (strength of the nearest-neighbour coupling) or purely non-Hermitian (strength of the non-Hermitian gauge field), both of which we connect in a phase transition equation.

We implement our ideas in an optical system with controllable dissipation, consisting of coupled optical fibre loops^11,34,35^, where the light propagation corresponds to the time evolution of a single-particle wavefunction within a one-dimensional discrete lattice. By modulating the phase of the light, we are able to emulate a quasicrystalline lattice potential. In the Hermitian aperiodic lattice, we observe a Floquet version of the Hofstadter butterfly quasienergy spectrum. When an imaginary gauge field is added to the quasicrystal, we observe a topological triple phase transition.

## Theory

We start by introducing a Floquet version of the Hermitian AAH model and derive its metal–insulator transition, as well as its energy spectrum at the phase transition point (that is, the Floquet Hofstadter butterfly). In a second step, we include a non-Hermitian skin effect modulation^11^ to observe the topological, PT symmetry-breaking and metal–insulator phase transitions.

**Fig. 2:**
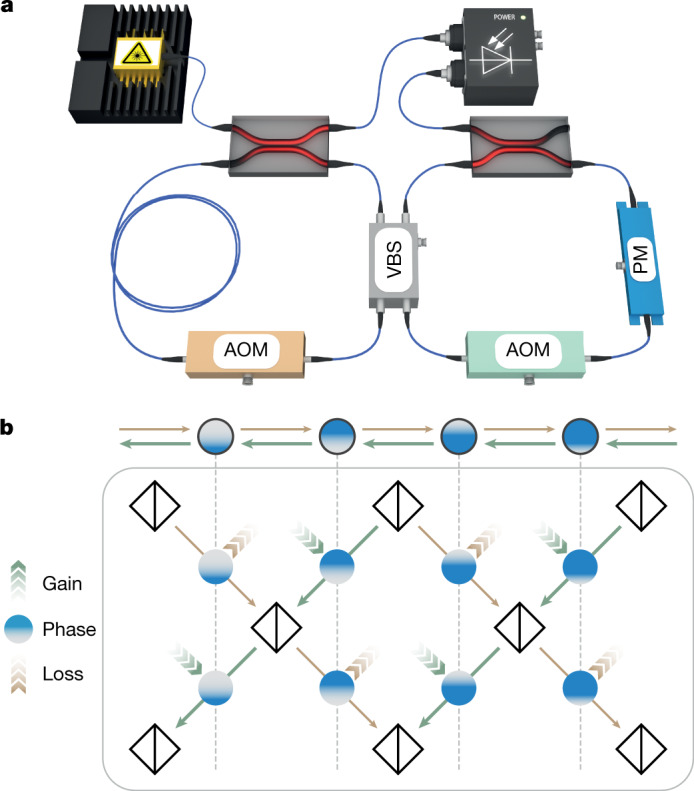
Experimental implementation of one-dimensional quasicrystals via photonic quantum walks. **a**, Simplified experimental setting for realizing photonic lattices via discrete-time quantum walks. Two unequally long optical fibre loops are connected by a variable beam splitter (VBS) that controls *β*. The non-Hermitian potential is realized by introducing controlled optical losses with acousto-optical modulators (AOMs). A phase modulator (PM) emulates the real part of the potential and creates the quasicrystalline order. Photodetectors measure the light intensity in both loops and hereby the time evolution of the quantum walk. **b**, The one-dimensional lattice (top) is implemented with a one-dimensional quantum walk (bottom) (equation (1)), based on a mesh lattice of beam splitters that is created with the coupled fibre loops (Supplementary Section 1). Gain and loss are incorporated at different lattice positions and in a two-step Floquet protocol, such that the skin effect modulation with anisotropic coupling with strength *h* (imaginary gauge field) is obtained. In a similar way, the potential (strength corresponds to amount of blue coloured filling) of the AAH model is realized via phase modulation (equation (2)) with a spatial phase gradient and alternating sign. The combined modulations realize the non-Hermitian Floquet AAH model based on a discrete-time quantum walk.

**Fig. 3:**
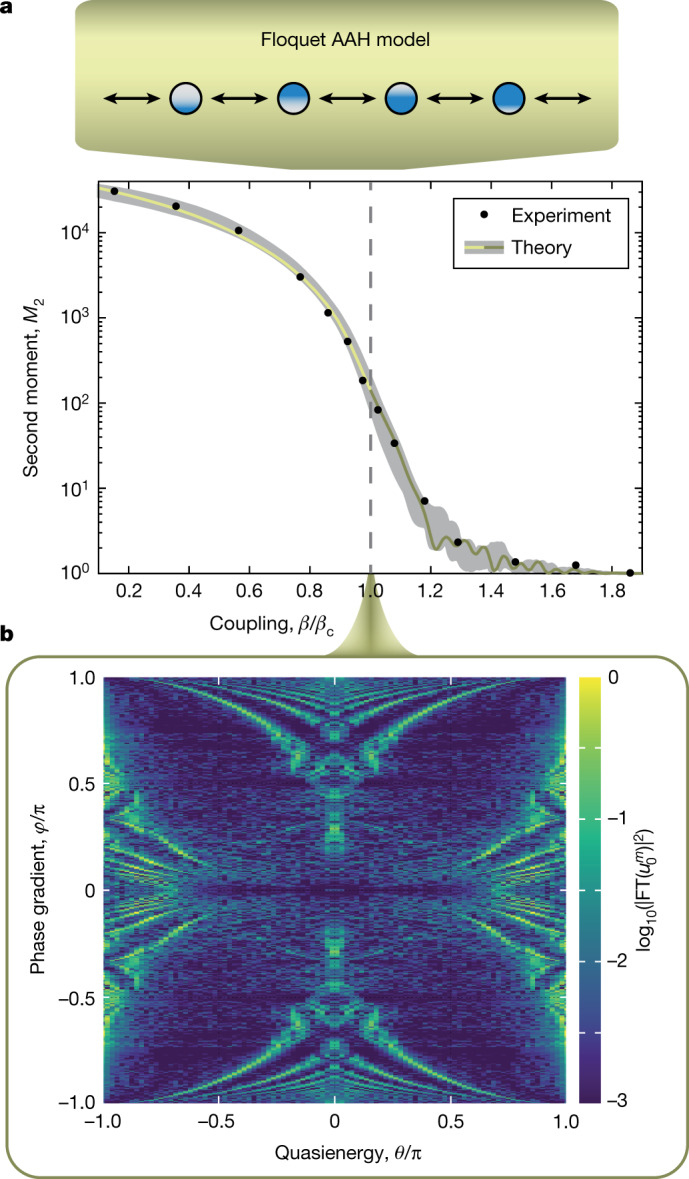
Experimental Floquet metal–insulator transition and Floquet Hofstadter butterfly in the AAH model. **a**, Upon a single-site excitation of the Hermitian quasicrystal, the spatial spreading of the wavefunction is shown via the second moment *M*_2_ of its position operator after a large propagation time of *m* = 200. The grey area marks the tolerance region of expected deviations owing to limited accuracy in the lattice parameters (Methods). One can see that a drastic spatial localization of the wavefunction sets in upon increasing the intersite coupling parameter beyond *β*_c_ = π/4 (top). This is exactly the metal–insulator phase transition, known from the static AAH model. **b**, At the symmetry point *β* = π/4, the Floquet Hofstadter–Harper model emerges. The evaluation of the quasienergies *θ* at *m* = 380 for 200 different phase gradients *φ* yields the Floquet Hofstadter butterfly (bottom) (Supplementary Section 3). The large propagation time allows for the high energy resolution. Compared with the original Hofstadter butterfly, our Floquet butterfly appears to be horizontally squeezed, due to the 2π periodicity in *θ*. The distribution of eigenvalues *θ* is obtained by applying the temporal Fourier transform (FT) to ${u}_{0}^{m}$. Here, $\left|{u}_{0}^{m}\right|$ is retrieved from the intensity measurement. The phase information, which is only a ± sign here, is lost in the intensity measurement, and we therefore added this minor information to the experimental data based on equation (1). The Floquet butterfly without this sign information is shown in Supplementary Section 3.

In its original form, the AAH model^22^ represents a chain of sites with an on-site potential that is incommensurable compared with the lattice spacing, thus realizing a quasicrystal^23^. We consider a Floquet implementation of the AAH model, where the static AAH model is mapped onto a discrete-time quantum walk^34,36^, as shown in Fig. 2. Such a quantum walk describes the evolution of a quantum particle on discrete lattice sites *n*, which are coupled to only the neighbouring sites, in a two-step protocol along the propagation direction *m* (time axis). The underlying lattice potential is controlled by introducing local phase terms between the couplings. The dynamics is governed by the equations

1 $${u}_{n}^{m+1}=({\cos (\beta )u}_{n+1}^{m}+{\rm{i}}\,\sin (\beta ){v}_{n+1}^{m}){{\rm{e}}}^{{\rm{i}}{\varphi }_{n}^{u}},$$

$${v}_{n}^{m+1}=({\rm{i}}\,\sin (\beta ){u}_{n-1}^{m}+{\cos (\beta )v}_{n-1}^{m}){{\rm{e}}}^{{\rm{i}}{\varphi }_{n}^{v}},$$

where ${u}_{n}^{m}$ denotes the amplitude on left-moving paths of the quantum walk and ${v}_{n}^{m}$ denotes the corresponding amplitude on right-moving paths at position *n* and time step *m* (Supplementary Section 1). We find that it is possible to implement a Floquet version of the AAH model by a temporally changing two-step phase modulation ${\varphi }_{n}^{u,v}$ and constant sublattice (nearest-neighbour) coupling *β*. This modulation reads

2 $${\varphi }_{n}^{u}={\left(-1\right)}^{m}\left(n+\frac{1}{2}\right)\frac{{\rm{\pi }}\phi }{2},\,{\varphi }_{n}^{v}={\left(-1\right)}^{m+1}\left(n-\frac{1}{2}\right)\frac{{\rm{\pi }}\phi }{2},$$

where *φ* is an irrational number for an incommensurable potential. The motivation for this modulation stems from the appearance of the vector potential in the original Harper model^26^, where the magnetic field in the Landau gauge leads to phase gradients with opposite signs, depending on the hopping direction. This is reflected by the opposite signs in the phase terms for the left- and right-moving components ${\varphi }_{n}^{u,v}$. The additional sign flip along the temporal direction *m* is based on the sublattice structure and assures that the same phase terms are aligned. This is illustrated in Fig. 2b, where the vertical lines connect identical phase modulations. Like in the original AAH model, our Floquet version displays a localization–delocalization phase transition without mobility edges, as the coupling parameter *β* is varied. The sudden metal–insulator phase transition is observed at the symmetry point *β* = π/4. This result can be rigorously proven from a self-similarity argument and it is also supported by a numerical analysis of the Lyapunov exponent (see Supplementary Section 2 for details). At this critical point, which lies exactly at the localization transition, when the phase gradient *φ* is varied, a Floquet version of the Harper–Hofstadter butterfly emerges for the quasienergy spectrum(Fig. 3b).

We now turn to the non-Hermitian extension of the AAH model. To this end, we employ a skin effect modulation^11^. The exact mapping is shown in Fig. 2, where, besides a phase modulation, the amplitudes are also manipulated. Mathematically, this corresponds to a complex phase term ${\varphi }_{n}^{v}\to {\varphi }_{n}^{v}-{\rm{i}}h$ and ${\varphi }_{n}^{u}\to {\varphi }_{n}^{u}+{\rm{i}}h$, which, in turn, leads to an effective anisotropic coupling^11^. The non-Hermitian contribution *h* to the phase term effectively corresponds to the implementation of an imaginary gauge field^28^ (see Supplementary Section 5).

**Fig. 4:**
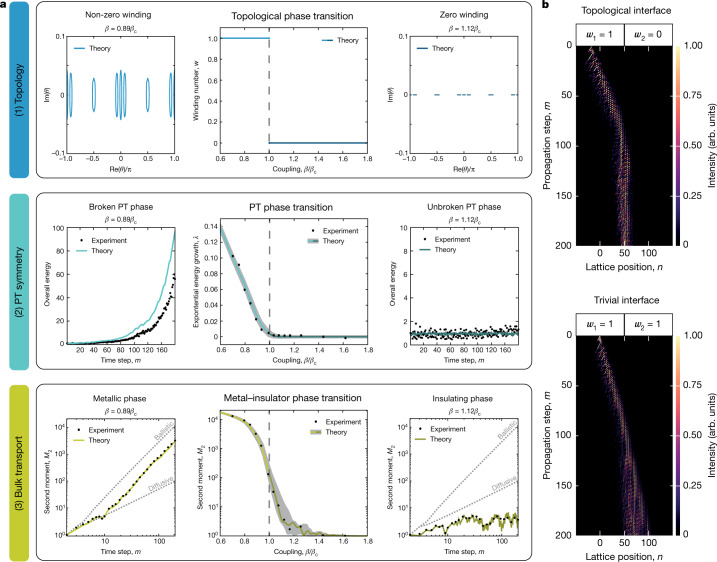
Experimental triple phase transition in a non-Hermitian Floquet quasicrystal. **a**, Three simultaneous phase transitions are shown from top to bottom. The two regimes of the triple phase transition are separated from left to right by the vertical dashed line. Top: for *β* < *β*_c_ (left), the quasicrystal is expected to be in a topologically non-trivial phase, owing to the formation of point-energy gaps with non-zero winding *w* = 1. The quasienergy spectra are obtained from numerical diagonalization of the Floquet propagator with periodic boundary conditions. For *β* > *β*_c_ (right), the topological phase changes, as the spectrum becomes real and the winding changes. Centre: for *β* < *β*_c_ (left), the quasicrystal is in the broken PT phase, which is marked by the exponential growth *λ* of the overall energy ${{\sum }_{n}|{u}_{n}^{m}|}^{2}+{|{v}_{n}^{m}|}^{2}\propto {{\rm{e}}}^{{\rm{\lambda }}m}$ in time. For *β* > *β*_c_ (right), the system changes to the unbroken PT phase, where the spectrum becomes real, and the overall energy becomes on-average constant. Bottom: for *β* < *β*_c_ (left), the quasicrystal is in the delocalized phase, which is marked by a monotonic increase of the second moment that indicates strong spatial spreading of the wavefunction. For *β* > *β*_c_ (right), all eigenstates become exponentially localized, which is marked by the extremely low and bounded second moment. All experiments are based on single-site excitations. The experimental data agree well with the predicted transition point at *β*_c_ = 0.275π. The grey areas mark the tolerance regions of expected deviations owing to limited accuracy in the lattice parameters (Methods). **b**, Although a direct measurement of the winding number is not possible with the experimental setup, we observe light localization at a topological interface (top, *β*_1_ = 0.70*β*_c_ and *β*_2_ = 1.03*β*_c_ compared with a trivial interface (bottom, *β*_1_ = 0.70*β*_c_ and *β*_2_ = 0.89*β*_c_), where light does not localize at the interface. The localization at the interface vanishes, as soon as the right medium exceeds critical coupling *β*_c_, such that both sides would have the same topological winding *w* = 1.

By superimposing the quasiperiodic phase potential with the non-Hermitian contribution, the non-Hermitian Floquet AAH model is formed. Our analysis shows that the non-Hermitian model exhibits a triple phase transition (Fig. 4a) at the critical point *β*_c_, which is related to the anisotropy strength (or imaginary gauge field) *h* by (Supplementary Section 3)

3 $$h=\log \left(1+\frac{1}{\cos ({\beta }_{{\rm{c}}})}-\frac{1}{\cos \left(\frac{\pi }{4}\right)}\right).$$

In the Hermitian limit *h* → 0, the critical point takes the value of *β*_c_ = π/4, according to the self-duality argument. Besides the localization transition, now situated at *β*_c_(*h*), we find PT symmetry breaking at *β*_c_, separating a regime with an exponential net energy growth (*β* < *β*_c_) from a regime where the energy exchange with the environment is on-average balanced (*β* > *β*_c_). A derivation of the PT symmetry is provided in Supplementary Section 6. The third transition that occurs at this point is a topological one. As the quasienergies of equation (1) form closed contours in the complex plane for *β* < *β*_c_, a topological winding number can be introduced^9^

4 $$w=\mathop{{\rm{l}}{\rm{i}}{\rm{m}}}\limits_{L\to {\rm{\infty }}}\frac{1}{2\pi {\rm{i}}}\mathop{\mathop{\int }\limits^{2\pi }}\limits_{0}\frac{{\rm{\partial }}}{{\rm{\partial }}{\rm{\vartheta }}}\log \,det\,\left[H\left(\frac{{\rm{\vartheta }}}{L},h\right)-{\mathbb{I}}\,{\theta }_{{\rm{B}}}\right]{\rm{d}}{\rm{\vartheta }},$$

which counts the number of times the complex spectral trajectory encircles a base point quasienergy *θ*_B_ when the phase *ϑ* is varied from zero to 2π. Here, *H* is the Hamiltonian with periodic boundary conditions and *L* is the overall number of sites (Supplementary Section 4). The phase *ϑ* adds to the phase modulation in the form ${\varphi }_{u}\to {\varphi }_{u}+\vartheta /(2L)$and ${\varphi }_{v}\to {\varphi }_{v}-\vartheta /\left(2L\right)$ (Supplementary Section 4). One can further conclude from equation (3) that the triple phase transition shows a fundamental duality between Hermitian and non-Hermitian parameters, as it can be induced by changing either the site coupling *β* or the non-Hermitian gauge field *h*. Owing to the non-Hermitian contributions, the symmetry protection of the critical point is lost, and the phase transition point can be tuned with the anisotropy strength.

## Experimental results

The Floquet non-Hermitian quasicrystal is implemented using an integrated photonic platform, in which classical light pulses propagate in two coupled and unequal long fibre loops connected by a beam splitter (Fig. 2). This setting realizes a discrete-time quantum walk (Supplementary Section 1) and has proven to serve as versatile platform for the implementation of Hermitian^37,38^ and non-Hermitian^11,35^ synthetic lattices.

In a first experiment, the properties of the Hermitian Floquet AAH model are probed, using the potential of equation (2). Upon exciting a single lattice site $({u}_{n}^{0},{v}_{n}^{0})=({\delta }_{n0},0)$, where *δ*_ij_ denotes the Kronecker delta, we measure the evolving intensity distribution ${|{u}_{n}^{m}|}^{2}+{|{v}_{n}^{m}|}^{2}$ of both loops. The localization transition is measured by evaluating the second moment ${M}_{2}={\sum }_{n}{n}^{2}({|{u}_{n}^{m}|}^{2}+{|{v}_{n}^{m}|}^{2})$of the wave packet after *m* = 200 propagation steps, while using the irrational lattice frequency of $\phi =\left(\sqrt{5}-1\right)/2$, which is the inverse of the golden mean. The dynamical wave packet spreading is evaluated for several different splitting ratios *β*, encompassing the phase transition region around *β* = π/4. The results clearly show the occurrence of a delocalization–localization transition (Fig. 3a), in agreement with the numerical predictions.

To highlight the feasibility and controllability of our photonic setting, in a second experiment we measure the quasienergy spectrum at the phase transition point (*β* = π/4) for different potential frequencies in the range $\phi \in \{0,{\rm{\pi }}\}$, that is, different magnetic fluxes in the corresponding Harper equation. To obtain the information about the energy spectrum, we apply a Fourier transform on the lattice-site amplitudes along the propagation direction *m* (see Supplementary Section 3 for details). By using a large propagation time of *m* = 380, a high energy resolution is obtained. For the experiments, the phase modulation equation (2) is slightly adapted to the form ${\varphi }_{n}^{u}={\left(-1\right)}^{m}n{\rm{\pi }}\phi$ and ${\varphi }_{n}^{v}=0$, which is equivalent to the used modulation, as the lattice symmetry and the relative phase difference between the *u* and *v* components is maintained. With the adapted phase potential, a single site excitation ${u}_{n}^{0}={\delta }_{n0}$ now results in ${u}_{0}^{m}\in {\mathbb{R}}$ owing to the underlying symmetry of the lattice, which is explained in Supplementary Section 3. In an intensity measurement, any phase information is lost. However, as ${u}_{0}^{m}\in {\mathbb{R}}$, only the information about the sign is lost, which makes the phase measurement less crucial for the reconstruction of the spectrum. In Fig. 3b, we show the Fourier transform of the experimentally retrieved $|{u}_{0}^{m}|$, complemented with the sign information obtained from our simulations. The reconstructed energy spectra are reminiscent of the famous Hofstadter butterfly with a fractal structure, clearly showing that our Floquet photonic quantum walk can well reproduce the rich features of the Hermitian AAH model.

In the last and central experiment, we consider the non-Hermitian quantum walk, which displays the triple phase transition. The imaginary gauge phase *h* can be continuously varied from zero (Hermitian limit) to about *h* ≈ 0.12. In the experiment, we fixed the gradient to the irrational Diophantine value $\phi =\left(\sqrt{5}-1\right)/2$. By varying the coupling *β* at a fixed non-vanishing gauge phase *h*, we simultaneously monitor the topology, energy exchange and bulk transport of the non-Hermitian quasicrystal (Fig. 4a). One can clearly see that the system undergoes three phase transitions as soon as *β* exceeds *β*_c_ = 1.1π/4, where all eigenvalues become real and the energy gaps in the complex plane are closed (Fig. 4a, top row). To verify the topological nature of this phase transition, we deduce the change of the winding number based on the dynamical (propagation) data, either in the bulk or by comparing a topologically non-trivial interface to a trivial interface (Fig. 4b). From the presence of a biased transport in the bulk owing to the non-Hermitian skin effect (as visible far from the interface), one can infer a non-zero winding^39^, that is, a topologically non-trivial phase. However, the skin effect is suppressed in the localized phase, from which one can deduce a zero winding, that is, a topologically trivial phase^9,39^. Furthermore, we numerically verified that only if the winding numbers of the adjacent media are different, light localizes at the interface instead of being transmitted through it. This observation supports the change of the topological phase, and such a behaviour suggests that the existing non-Hermitian bulk–boundary correspondence^9,40^ might be extendable to quasicrystals. Simultaneously, at the phase transition point, the exponential accumulation of energy in the systems stops, and the overall energy becomes on-average conserved as the PT phase transition from the broken to the unbroken PT phase occurs (Fig. 4a, centre row). In addition, a localization transition is observed: for *β* < *β*_c_, there is a strong growth of the wavefunction’s second moment, whereas for *β* > *β*_c_, the second moment becomes bounded, indicating that all eigenstates are localized (Fig. 4a, bottom row).

Our experimental results show that all three phase transitions coincide at *β*_c_(*h*). We stress that, although in the Hermitian limit only a localization–delocalization phase transition can be found at *β*_c_ = π/4, in the non-Hermitian case a triple phase transition emerges, where the critical point shifts to *β*_c_ = 1.1π/4, in agreement with our theoretical analysis.

## Conclusion

We have experimentally demonstrated the concurrence of a triple phase transition in a one-dimensional non-Hermitian synthetic quasicrystal, which is realized in a Floquet photonic quantum walk with a controlled imaginary gauge field and an quasicrystalline potential. The usual metal–insulator phase transition found in the Hermitian limit corresponds to the simultaneous breaking of PT symmetry and to a topological phase transition when a synthetic imaginary gauge field is applied to the quasicrystal. Our results provide experimental evidence on the exceptional properties of synthetic non-Hermitian quasicrystalline matter in terms of topology, localization–delocalization and symmetry-breaking phase transitions, which are responsible for phenomena that are drastically distinct from the familiar Hermitian realm. As such, our results have far-reaching consequences in a wide range of energy-conserving and open systems, as it offers a step towards unifying seemingly distinct phenomena.

## Methods

### Experimental setup

The experimental setup is similar to the setup described in refs. ^11,35^. The setup consists of two optical fibre loops, which are coupled by a variable beam splitter that controls the coupling parameter *β*. The loops are of unequal length, such that the roundtrip times are approximately given by 27 μs ± 50 ns. The time difference of 100 ns defines the temporal width of a time-bin, in which the lattice positions *n* are encoded, such that approximately 270 positions can be encoded in the loops. The extended propagation time in each loop is achieved by using spools of single mode fibre (Corning Vascade LEAF EP). At the beginning of each measurement, a single 70-ns pulse is injected into the longer loop, here called the v-loop, via a fused fibre-optical beam splitter. The initial pulse is generated with a continuous-wave distributed feedback laser (JDS Uniphase, 1,550 nm) in combination with a Mach–Zehnder modulator (SDL Integrated Optics), which cuts out 70-ns pulses via intensity modulation. A pulse-picker acousto-optical modulator (Gooch & Housego) is used to further increase the on–off ratio of the light intensity. After the initial injection, the pulse circulates in the loop arrangement and periodically splits up at the variable beam splitter and multipath interference between the emerging subpulses takes place. The time multiplexing imposes the interference condition that two pulses will interfere only if they have travelled a permutation of the same sequence of long and short loop roundtrips. This interference condition guarantees an extremely stable phase relation between the interfering pulses, as external phase noise is acquired equally for a large frequency range of noise. The temporal intensity distribution of the propagating pulses is obtained by photodetectors (Thorlabs). The output voltages of the photodetectors are amplified with a logarithmic amplifier (FEMTO HLVA-100) and then acquired by an oscilloscope (R&S RTO1104). With the propagation timescales Δ*t* = 100 ns and *T* =27 μs, one can map the light intensity onto the discrete 1 + 1D lattice (time step *m* and position *n*). The measured pulse intensities correspond to the squared modulus of the wave function at lattice site *n* and time step *m*. To realize desired the phase and gain/loss modulation, an additional phase modulator (ixBlue Photonics) is placed in the u-loop and an acousto-optical amplitude modulator (zeroth order, Brimrose) is placed in each loop. To also realize gain and compensate for global losses (for example, insertion loss or propagation losses in optical fibre) an erbium-doped fibre amplifier (Thorlabs) is placed in each loop. The amplifiers are optically gain clamped with by an additional distributed feedback laser (JDS Uniphase, 1,538 nm) that is coupled to the amplifier input via wavelength division multiplexing coupler (AC Photonics). Excess light from the gain clamping protocol is removed by an optical band-pass filter (WL Photonics), which also suppresses optical noise that stems from the amplification. All optical components are designed for operation at 1,550-nm wavelength and use a standard single mode fibre (SMF28 or comparable). The polarization is aligned between each loop and in front of polarization-sensitive components. Arbitrary waveform generators (Keysight Technologies, 33622A) generate the voltage signals that drive the electro-optical modulators. For each measurement, we perform an additional noise measurement in which no input pulse is injected. The measured light intensity then corresponds to the noise data and can be subtracted from the original date in post-processing.

### Energy growth estimation

In the broken PT phase, it is expected that the overall light intensity can exponentially grow with propagation time *m*. Such growth in optical power can lead to a quick and nonlinear gain saturation of the amplifiers. Furthermore, high optical power might induce nonlinear effects via self-phase modulation or damage the optical components. To avoid the exponential light intensity growth, we impose artificial losses to the system in the broken phase, until the overall power no longer grows exponentially. These losses are equal in both loops and do not vary in time, such that the overall dynamic in the quantum walk is not affected. To do so, we decrease the gain of both amplifiers, such that an excess net loss is induced in each roundtrip. Afterwards, we keep these parameters and measure a Hermitian quantum walk as a control measurement. Owing to the excess loss, the overall light intensity of the Hermitian quantum walk is not constant, but exponentially decays with propagation time *m*. From this decay, one can deduce the power growth of the previously measured non-Hermitian system.

### Experimental error and tolerance regions

The experimental error is captured via the systematic and statistical errors. The tolerance regions show the expected deviations owing to systematic errors. We assume that the main contribution for systematic errors stems from the limited precision of the experimentally realized lattice parameters. The lattice parameters are implemented via phase and amplitude modulation of the propagating light and via the coupling of the variable beam splitter that connects the fibre loops. Therefore, the systematic error stems mainly from the limited precision of the electro-optic driving, for instance, owing to bias drifts and tolerances in the look-up curves of the modulators. We assume a relative error of ±1% in the modulation parameters (that is, the imprinted phases, gain/loss and the coupling *β*) and estimate the resulting error for the second moments and the energy growth via error propagation. The observed statistical fluctuations on repeating individual single-site excitations at least 10 times were negligible compared with the systematic error. The resulting overall errors can explain most of the discrepancies between the experimental and the theoretical data.

### Accuracy of the irrational phase gradient

The experimental realization of the Hermitian and non-Hermitian Floquet AAH model required the phase gradient strength *φ* to be an irrational number to realize a potential that is incommensurable with respect to the lattice site spacing. It is therefore natural to ask with what accuracy an irrational parameter can be achieved and how any rational number (finite size) approximation would affect the results. On the one hand, the effect of a limited accuracy is already captured within the grey areas in Figs. 3, 4. Therefore, one can conclude that the limited accuracy of the phase modulation does not prevent an observation of the phase transitions that is based on the propagation data. On the other hand, it should be noted that owing to the time–energy uncertainty principle, in an experiment one can never resolve with an arbitrarily high precision the fine spectral or dynamical features observed when approaching the irrational *φ*. To clarify this point, let us assume that *T* is the largest observation time (time step) of the dynamics, and that we excite the lattice at the single site *n* = 0. The excitation spreads in the lattice with an upper speed bound *v* ≤ 1 and thereby it is clear that one never probes more than *L* = *vT* = *T* sites in the lattice at the left and the right sides from *n* = 0. Therefore, the experiment would yield the same results for two different values of the phase gradients *φ*_1_ and *φ*_2_ such that d*φ* = |*φ*_2_ − *φ*_1_| is of the order of (or smaller than) 1/*L* = 1/*T*, because the light pulses effectively probe the same potential over the spatial interval *L*. Hence, in the experiment, we cannot distinguish any finer spectral or dynamical features arising from any change of d*φ* smaller than 1/*T*, and this also sets the accuracy we require to achieve the target irrational value of $\phi =\left(\sqrt{5}-1\right)/2.$

## Online content

Any methods, additional references, Nature Research reporting summaries, source data, extended data, supplementary information, acknowledgements, peer review information; details of author contributions and competing interests; and statements of data and code availability are available at 10.1038/s41586-021-04253-0.

## Supplementary information

Supplementary Information This file contains Supplementary Notes 1–5, including Supplementary Figs. 1–4 and additional references.

## Data Availability

All experimental data that have been used to produce the results reported in this manuscript are available in an open-access data repository^41^.
