# Supplementary material for: Topological triple phase transition in non-Hermitian Floquet quasicrystals
Source: Nature. 2022 Jan 19;601(7893):354–9. doi: 10.1038/s41586-021-04253-0 (PMC8770143; doi:10.1038/s41586-021-04253-0)
Supplement: Supplementary file 1 — This file contains Supplementary Notes 1–5, including Supplementary Figs. 1–4 and additional references. [file 41586_2021_4253_MOESM1_ESM.pdf]

nature portfolio

https://doi.org/10.1038/s41586-021-04253-0

Supplementary information

Topological triple phase transition in non-Hermitian Floquet quasicrystals

In the format provided by the authors and unedited

Topological triple phase transition in non-Hermitian Floquet quasicrystals

Supplementary Information

Sebastian Weidemann[1,4], Mark Kremer[1,4], Stefano Longhi[2,3] and Alexander Szameit[1]

Institute for Physics, University Rostock, Albert-Einstein-Straße 23, 18059 Rostock, Germany.

[2]Dipartimento di Fisica, Politecnico di Milano, Piazza Leonardo da Vinci 32, 20133 Milano, Italy.

[3]IFISC (UIB-CSIC), Instituto de Fisica Interdisciplinar y Sistemas Complejos - Palma de Mallorca, Spain.

[4]These authors contributed equally to this work.

Six additional notes are included. Section S1 describes the time-multiplexed photonic quantum walk setup that realizes a non-Hermitian synthetic quasicrystal. In section S2 the metal-insulator phase transition of the Floquet Aubry-André-Harper model is derived, based on a self-duality argument, and results on Lyapunov exponent calculation (inverse of localization length) are presented. Section S3 deals with the Fourier transform method used to detect the Floquet Hofstadter butterfly (quasienergy spectrum) from wave spreading measurements in the Hermitian lattice. Finally, sections S4 and S5 discuss the non-Hermitian delocalization transition induced by the imaginary gauge field and the hidden PT symmetry of the model, respectively.

1 Time-multiplexed quantum walks

In this section, the mapping of the light propagation in two coupled optical fibre loops to the light propagation through a photonic mesh lattice (discrete-time quantum walk) is discussed. Further, we briefly discuss why it is possible to realize ‘quantum’ walks with classical light and while using optical amplification. The idea is that the propagation through a photonic mesh lattice can be implemented in a much simpler arrangement of two coupled optical fibre loops, via time-multiplexing (temporal encoding). Time-multiplexed fibre loop lattices have proven to be a powerful experimental platform[1–4]. To understand the concept, let us consider two unequally long optical fibre loops that are connected by a beam splitter. A single optical pulse is injected into the longer loop. By comparing the light evolution in the loops with the light evolution in a mesh lattice of beam splitters, one can see that the loops temporally encode the beam splitter lattice (Fig. S1).

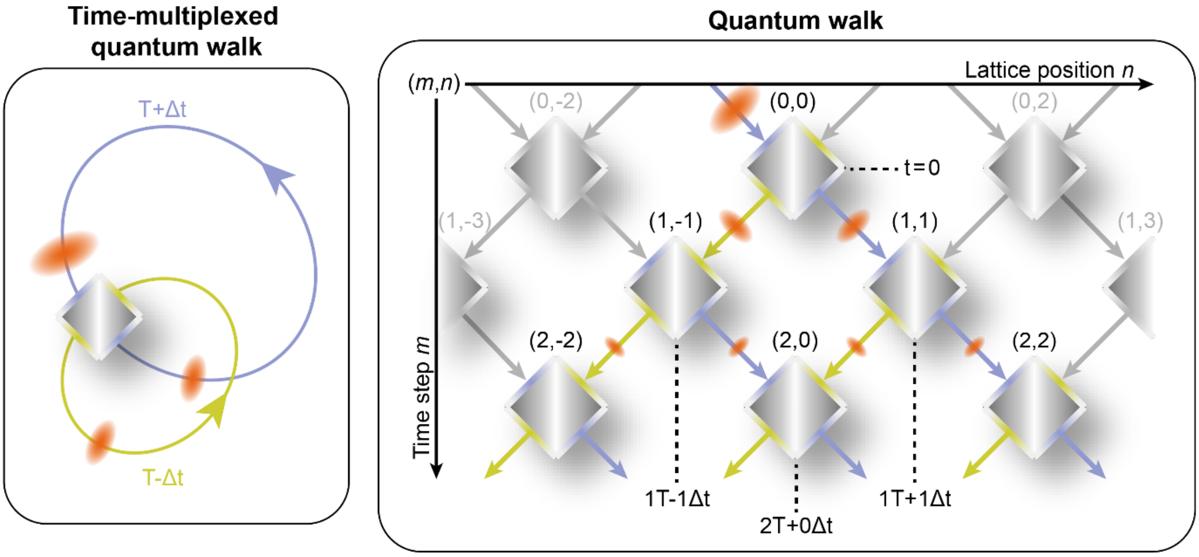

Figure S1 | Time-multiplexed quantum walks. The pulse propagation in fibre loops (left panel) temporally encodes the quantum walk on the line (right panel). After the injection, the initial pulse (red-filled circle) splits up into two pulses, which return to the splitter with a time difference of $2\Delta t$. This temporal separation can be regarded as an arrival at two spatially separated splitters as shown on the right. After the next roundtrip, two pulses will meet and interfere at the splitter, because they have the same arrival time. The resulting interplay of temporal delays and multipath-interference forms a 1+1D beam splitter lattice (synthetic mesh lattice). The discrete positional sites n and discrete time steps m in the mesh lattice are encoded in time scales Δt and T of the fibre system, respectively.

Assuming a beam splitter with coupling $\beta \in [0, \pi/2]$, the lattice dynamics for the pulse amplitudes u_n^m and v_n^m in the short and long loop, respectively, is described by the discrete-time coupled equations

$$\begin{aligned} u_n^{m+1} &= \cos(\beta)\, u_{n+1}^m + i \sin(\beta)\, v_{n+1}^m, \\ v_n^{m+1} &= i \sin(\beta)\, u_{n-1}^m + \cos(\beta)\, v_{n-1}^m. \end{aligned} \tag{1}$$

By adding amplitude modulators and a phase modulator into the loops, equations (1) of the SI are modified to equations (1) of the main text. In our experiment, the system is probed with a classical light from a (pulsed) single-mode laser source, each pulse carrying a large number of photons. From the perspective of quantum optics, a single-particle quantum walk can be captured with classical light (a coherent state)[5,6]. The probability to detect a single photon at the various sites of the network, after some propagation length, is the same as the ‘classical’ intensity distribution observed when the network is excited by a coherent state carrying an arbitrary number of photons (like the light emitted by a single-mode laser). Moreover, classical light can be coherently amplified in erbium-doped amplifiers. Hence, all the relevant information for the interference processes, i.e. amplitude and phase, are reproduced via stimulated emission in the amplifier. Owing to such an equivalence, the wording “photonic quantum walk” is justified for the single-particle quantum walks and widely adopted in the open literature.

2 Metal-insulator transition in the Floquet Aubry-André-Harper model

In this section, we derive and analyze the metal-insulator transition of the Hermitian limit of the Floquet quasicrystal. The analysis reveals that in the Hermitian limit our model indeed resembles a Floquet implementation of the Aubry-André-Harper model, as its characteristic features are captured. These include the independence of Lyapunov exponents from energy and the sudden transition with all wave functions being extended to being localized, without mobility edges, as β is varied across a critical point. A semi-analytical expression for the localization length of wave functions is also derived, which is confirmed numerically by direct Lyapunov exponent computation. The discrete-time evolution for the quantum walk is captured by the propagator U over the full Floquet period, which involves two time steps m. In the first time step one has

$$u_n^{(m+1)} = \lambda\Big(\cos(\beta)\, u_{n+1}^{(m)} + i \sin(\beta)\, v_{n+1}^{(m)}\Big) \exp(i\phi_n^u),$$

$$v_n^{(m+1)} = \frac{1}{\lambda}\Big(i \sin(\beta)\, u_{n-1}^{(m)} + \cos(\beta)\, v_{n-1}^{(m)}\Big) \exp(i\phi_n^v), \tag{2}$$

while in the second time step one has

$$
\begin{aligned}
u_n^{(m+2)} &= \lambda\Big(\cos(\beta)\, u_{n+1}^{(m+1)} + i \sin(\beta)\, v_{n+1}^{(m+1)}\Big) \exp(-i\phi_n^u)\,, \\
v_n^{(m+2)} &= \frac{1}{\lambda}\Big(i \sin(\beta)\, u_{n-1}^{(m+1)} + \cos(\beta)\, v_{n-1}^{(m+1)}\Big) \exp(-i\phi_n^v).
\end{aligned}
\tag{3}
$$

In the above equations, we have set

$$
\phi_n^u = \left(n + \frac{1}{2}\right)\frac{\pi\varphi}{2}\,, \qquad \phi_n^v = \left(-n + \frac{1}{2}\right)\frac{\pi\varphi}{2}
\tag{4}
$$

while $\lambda = \exp(-h)$ is the impressed gain/loss modulation at each step. Here we focus our attention to the Hermitian limit, which is obtained by $h = 0$. By introducing the discrete Fourier transform

$$
U_l^{(m)} = \sum_n u_n^{(m)} \exp(-iln\phi)\,, \qquad V_l^{(m)} = \sum_n v_n^{(m)} \exp(-iln\phi)
\tag{5}
$$

with $\phi = \varphi\pi/2$, it can be readily shown that in the dual (Fourier) space the amplitudes $U_l^{(m)}, V_l^{(m)}$ satisfy the two-step maps defined by

$$
\begin{aligned}
U_l^{(m+1)} &= \Big(\cos(\beta)\, U_{l-1}^{(m)} + i \sin(\beta)\, V_{l-1}^{(m)}\Big) \exp(-i\phi_l^v)\,, \\
V_l^{(m+1)} &= \Big(i \sin(\beta)\, U_{l+1}^{(m)} + \cos(\beta)\, V_{l+1}^{(m)}\Big) \exp(-i\phi_l^u),
\end{aligned}
\tag{6}
$$

and

$$
\begin{aligned}
U_l^{(m+2)} &= \Big(\cos(\beta)\, U_{l+1}^{(m+1)} + i \sin(\beta)\, V_{l+1}^{(m+1)}\Big) \exp(-i\phi_l^u)\,, \\
V_l^{(m+2)} &= \Big(i \sin(\beta)\, U_{l-1}^{(m+1)} + \cos(\beta)\, V_{l-1}^{(m+1)}\Big) \exp(-i\phi_l^v).
\end{aligned}
\tag{7}
$$

The quasienergies θ are obtained from the eigenvalues $E = \exp(-i\theta)$ of the two-step propagator, either in physical (direct) or Fourier (dual) space. Note that an extended (localized)

wave function in physical space corresponds to a localized (extended) wave function in the Fourier space, and vice versa. In real space, the propagator has the form

$$U = \begin{pmatrix} \cos^2(\beta)\, \mathcal{A}_+^- \mathcal{A}_+^+ - \sin^2(\beta)\, \mathcal{A}_+^- \mathcal{A}_-^- & i \sin(\beta) \cos(\beta)\, (\mathcal{A}_+^- \mathcal{A}_-^- + \mathcal{A}_+^+ \mathcal{A}_+^-) \\ i \sin(\beta) \cos(\beta)\, (\mathcal{A}_-^+ \mathcal{A}_-^- + \mathcal{A}_-^+ \mathcal{A}_+^+) & \cos^2(\beta)\, \mathcal{A}_-^+ \mathcal{A}_-^- - \sin^2(\beta)\, \mathcal{A}_-^+ \mathcal{A}_+^+ \end{pmatrix} \quad (8)$$

while in dual (Fourier) space the two-time-step propagator reads (after shifting by half-period in discrete time and interchanging U_l and V_l)

$$M = \begin{pmatrix} \cos^2(\beta)\, \mathcal{A}_+^- \mathcal{A}_-^- - \sin^2(\beta)\, \mathcal{A}_+^- \mathcal{A}_+^+ & i \sin(\beta) \cos(\beta)\, (\mathcal{A}_+^- \mathcal{A}_-^- + \mathcal{A}_+^+ \mathcal{A}_+^-) \\ i \sin(\beta) \cos(\beta)\, (\mathcal{A}_-^+ \mathcal{A}_-^- + \mathcal{A}_-^+ \mathcal{A}_+^+) & \cos^2(\beta)\, \mathcal{A}_-^+ \mathcal{A}_+^+ - \sin^2(\beta)\, \mathcal{A}_-^+ \mathcal{A}_-^- \end{pmatrix}. \quad (9)$$

Here $\mathcal{A}_+^{\pm}$ are the matrices with non-vanishing elements $\exp(\pm in\phi)$ on the first upper diagonal (with respect to the main diagonal), while $\mathcal{A}_-^{\pm}$ are the matrices with non-vanishing elements $\exp(\pm in\phi)$ on the first lower diagonal. Moreover, the matrices satisfy the condition $\mathcal{A}_+^- = (\mathcal{A}_+^+)^*$ and $\mathcal{A}_-^- = (\mathcal{A}_-^+)^*$. From the form of U and M it readily follows that if E is an eigenvalue of the propagator U for a given coupling β, then $-E$ is an eigenvalue of M, when β is replaced by $-\beta + \pi/2$. For the symmetric coupling $\beta = \pi/4$ one has $\beta = -\beta + \pi/2$ (self-dual point) and therefore it follows that all eigenvectors of U (and M) are critical, i.e. they are neither extended nor localized (the quasienergy spectrum is singular continuous). This is the critical point known from the AAH model[7] and it is also originating from a self-duality argument. An analysis of the inverse participation ratio (IPR) of the numerically computed eigenvectors shows, that for $\beta < \pi/4$ all eigenvectors of U are extended, while for $\beta > \pi/4$ all eigenvectors are exponentially localized. Interestingly, the numerical analysis reveals that, like in the Aubry-André-Harper model, in the localized (insulating) phase all wave functions have the same localization length $1/\gamma$, which is very well approximated by the relation

$$\gamma = \log\left(1 + \frac{1}{\cos(\beta)} - \frac{1}{\cos(\pi/4)}\right). \quad (10)$$

A direct numerical computation of the Lyapunov exponent confirms the form (10) for the inverse of the localization length γ. In order to properly implement a recursive equation for the numerical calculation of the Lyapunov, one needs to take into account not only the temporal double-forward time step, but also the double-backward step. The reason behind this is that the eigenvalues of

the double-forward step is not just the sum of the eigenvalues of the individual single time steps. The double-forward time step reads:

$$\begin{aligned} u_n^{m+2} &= \big(\cos(\beta)\,[\cos(\beta)\,u_{n+2}^m + \mathrm{i}\sin(\beta)\,v_{n+2}^m]\mathrm{e}^{-\mathrm{i}\phi_{n+1}^u} \\ &\qquad + \mathrm{i}\sin(\beta)\,[\mathrm{i}\sin(\beta)\,u_n^m + \cos(\beta)\,v_n^m]\mathrm{e}^{-\mathrm{i}\phi_{n+1}^v}\big)\mathrm{e}^{\mathrm{i}\phi_n^u}, \\ v_n^{m+2} &= \big(\mathrm{i}\sin(\beta)\,[\cos(\beta)\,u_n^m + \mathrm{i}\sin(\beta)\,v_n^m]\mathrm{e}^{-\mathrm{i}\phi_{n-1}^u} \\ &\qquad + \cos(\beta)\,[\mathrm{i}\sin(\beta)\,u_{n-2}^m + \cos(\beta)\,v_{n-2}^m]\mathrm{e}^{-\mathrm{i}\phi_{n-1}^v}\big)\mathrm{e}^{\mathrm{i}\phi_n^v}. \end{aligned} \tag{11}$$

For the double backward step in reversed order (since the conjugate transpose operation † changes the order) one has

$$\begin{aligned} u_n^{m-2} &= \cos(\beta)\,\big[\cos(\beta)\,u_{n-2}^m\mathrm{e}^{-\mathrm{i}\phi_{n-2}^u} - \mathrm{i}\sin(\beta)\,v_n^m\mathrm{e}^{-\mathrm{i}\phi_n^v}\big]\mathrm{e}^{\mathrm{i}\phi_{n-1}^u} \\ &\qquad - \mathrm{i}\sin(\beta)\,\big[-\mathrm{i}\sin(\beta)\,u_n^m\mathrm{e}^{-\mathrm{i}\phi_n^u} + \cos(\beta)\,v_{n+2}^m\mathrm{e}^{-\mathrm{i}\phi_{n+2}^v}\big]e^{\mathrm{i}\phi_{n+1}^v}, \\ v_n^{m-2} &= -\mathrm{i}\sin(\beta)\,\big[\cos(\beta)\,u_{n-2}^m\mathrm{e}^{-\mathrm{i}\phi_{n-2}^u} - \mathrm{i}\sin(\beta)\,v_n^m\mathrm{e}^{-\mathrm{i}\phi_n^v}\big]\mathrm{e}^{\mathrm{i}\phi_{n-1}^u} \\ &\qquad + \cos(\beta)\,\big[-\mathrm{i}\sin(\beta)\,u_n^m\mathrm{e}^{-\mathrm{i}\phi_n^u} + \cos(\beta)\,v_{n+2}^m\mathrm{e}^{-\mathrm{i}\phi_{n+2}^v}\big]\mathrm{e}^{\mathrm{i}\phi_{n+1}^v}. \end{aligned} \tag{12}$$

Using a Floquet ansatz $(u_n^m, v_n^m) = (u_n, v_n)\mathrm{e}^{-\mathrm{i}\theta m/2} = \vec{\Psi}_n\mathrm{e}^{-\mathrm{i}\theta m/2}$ and the property, that $\phi_{n-1}^u = -\phi_n^v$ one gets the following matrix equation.

$$\begin{aligned} &2\cos(\theta)\vec{\Psi}_n \\ &= \begin{pmatrix} \cos^2(\beta)\mathrm{e}^{-\frac{\mathrm{i}\pi\varphi}{2}} & 2\sin(\beta)\cos(\beta)\sin\left(\frac{\pi\varphi}{2}\right) \\ 0 & \cos^2(\beta)\mathrm{e}^{\frac{\mathrm{i}\pi\varphi}{2}} \end{pmatrix}\vec{\Psi}_{n+2} \\ &+ \begin{pmatrix} \cos^2(\beta)\mathrm{e}^{\frac{\mathrm{i}\pi\varphi}{2}} & 0 \\ 2\sin(\beta)\cos(\beta)\sin\left(\frac{\pi\varphi}{2}\right) & \cos^2(\beta)\mathrm{e}^{-\frac{\mathrm{i}\pi\varphi}{2}} \end{pmatrix}\vec{\Psi}_{n-2} \\ &- 2\begin{pmatrix} \sin^2(\beta)\cos\big((n+1/2)\pi\varphi\big) & \sin(\beta)\cos(\beta)\sin\left(\frac{\pi\varphi}{2}\right)\mathrm{e}^{\mathrm{i}\pi\varphi n} \\ \sin(\beta)\cos(\beta)\sin\left(\frac{\pi\varphi}{2}\right)\mathrm{e}^{-\mathrm{i}\pi\varphi n} & \sin^2(\beta)\cos\big((n-1/2)\pi\varphi\big) \end{pmatrix}\vec{\Psi}_n \end{aligned} \tag{13}$$

This allows to calculate the Lyapunov exponent γ on all occupied (even) sites, by starting with an initial condition $\vec{\Psi}_0 = (0,0)$ and $\vec{\Psi}_2 = (1,1)$. The equation for the Lyapunov exponent reads

$$\gamma = \lim_{N\to\infty} \frac{1}{N} \sum_{n=1}^{N} \log\left(\frac{|\vec{\Psi}_{2n+2}|}{|\vec{\Psi}_{2n}|}\right), \tag{14}$$

with N being the overall number of sites. The numerical results indicate that the Lyapunov exponent γ does not depend on the value of θ, provided that it is assumed to belong to the quasienergy spectrum, and that γ vanishes for $\beta < \pi/4$. The behavior of the Lyapunov versus the coupling ratio β is shown in Fig. S2, together with the theoretical relation (10), showing a good agreement.

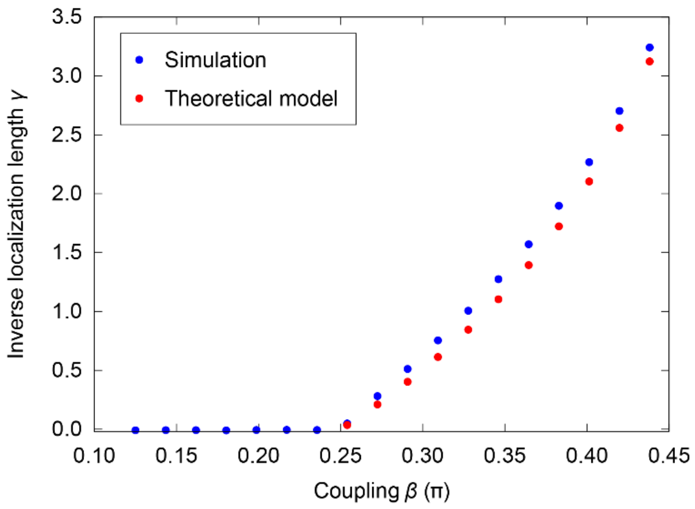

Fig. S2 | Localization length and Lyapunov exponent in the Floquet AAH model. Behaviour of Lyapunov exponent γ versus coupling constant β as obtained from Eq.(14) (blue) and from the theoretical equation (10) (red). Note that γ from changes from zero to non-zero, which indicates the localization transition from extended states ($\beta < \pi/4$) to localized states ($\beta \geq \pi/4$). The inverse of the localization length, predicted by Eq. (10) (red), agrees well with the numerical results of Lyapunov exponent computation.

3 Floquet Hofstadter butterfly: Quasienergy spectrum and Fourier transform (Hermitian lattice)

In this section, we provide details on the relation between the Floquet Hofstadter butterfly and the Fourier transform, which was used to obtain the corresponding quasienergy spectrum from the dynamical evolution measurements. We further show the experimental data without the numerical sign information of the phase of the lattice site amplitudes and compare all data with the theoretical predictions. In the end, we provide the energy spectrum obtained by numerical diagonalization of the Hamiltonian, to corroborate the effectiveness of the employed Fourier method.

The quasienergy spectrum θ of the time-discrete model, defined by equations (2-3) in SI with $h = 0$, can be obtained from the Floquet-Ansatz

$$\begin{pmatrix} u_n^m \\ v_n^m \end{pmatrix} = e^{-i\theta\frac{m}{2}} \begin{pmatrix} U_n \\ V_n \end{pmatrix}, \tag{15}$$

where the lattice periodic in time with a full Floquet period spans over two time steps m. The Floquet nature of the system allows to obtain the quasienergies θ directly by calculating the Fourier transform of u_n^m or v_n^m along the time axis m, starting from a single-site excitation. In the experiments, a measurement of the light intensity in the shorter loop corresponds to $|u_n^m|^2$. Hence, any phase information is lost in the intensity measurement. However, one can overcome this obstacle by relying on the symmetry properties of the system. Upon the single-site excitation $u_n^0 = \delta_{n0}$ the field u_0^m remains entirely real. Following this line of reasoning, one loses only the sign of the fields by transforming the square root of the measured intensities. The theoretical and experimental results are shown in Fig. S3.

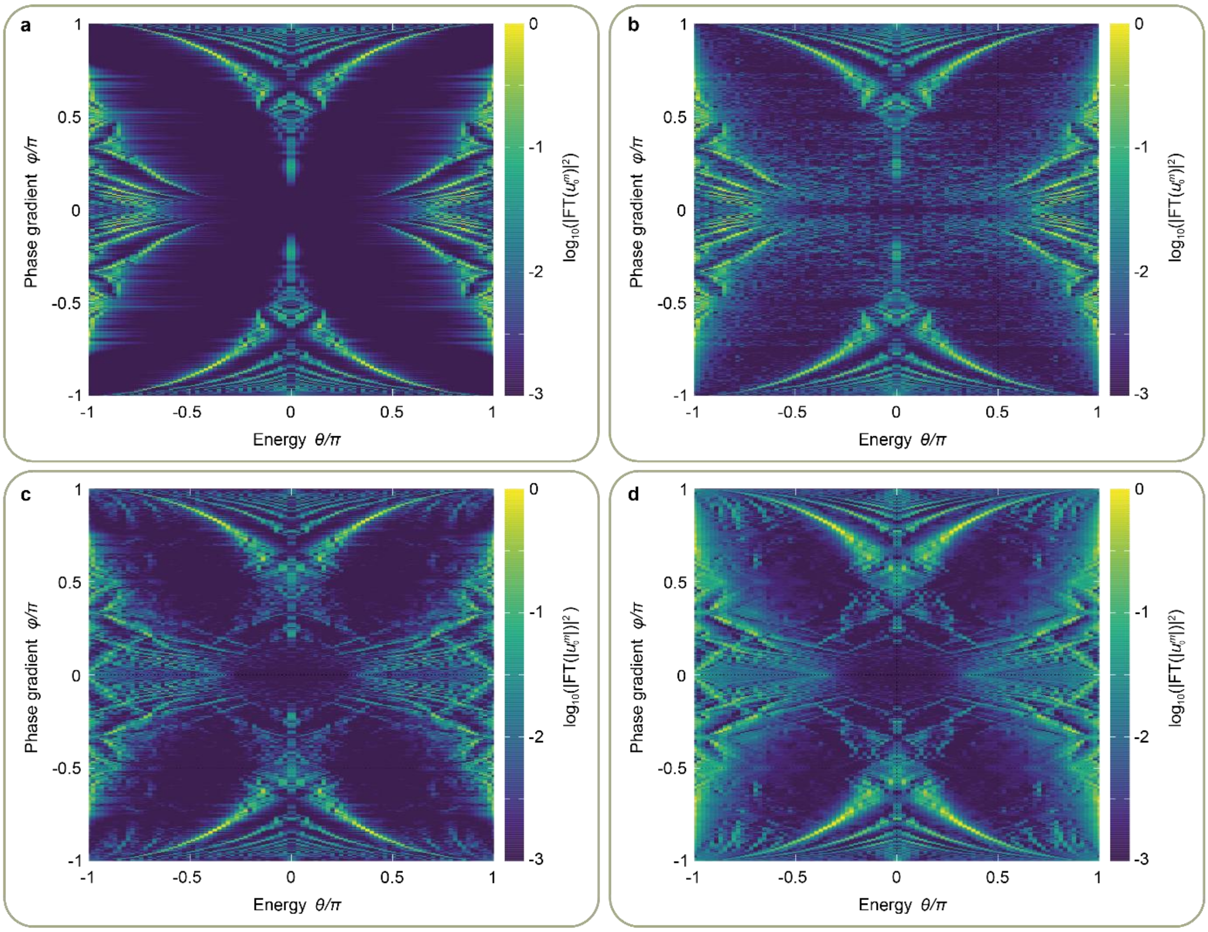

Fig. S3 | Floquet Hofstadter butterfly with and without phase information. a, The theoretical quasienergy spectrum of the Floquet Hofstadter butterfly, obtained by the Fourier transform of $u_0^m \in \mathbb{R}$ along the time-axis m at the central site $n = 0$ after 380 time steps of propagation and for 200 different phase gradients φ. **b**, The corresponding experimental data, where $|u_0^m|$ is measured and the numerically-computed sign information (phase) is added before applying the Fourier transform. **c,** The theoretical energy spectrum of the Floquet Hofstadter butterfly, without the phase information, obtained by Fourier transform of $|u_0^m|$. **d,** The corresponding experimental data, i.e., when the light intensity is processed without adding the numerically computed phase information. Note that the data for the negative gradients $\varphi < 0$ are mirrored from $\varphi > 0$, because these regions simply differ by a gauge transformation. The logarithmic scale of the colormap was chosen to enhance the visibility of even smallest populations of the corresponding Floquet eigenmodes, in order to reconstruct the spectrum. However, at the same time, even extremely small deviations between the spectrum with and without sign-information are greatly exaggerated.

To further corroborate the effectiveness of the Fourier method to determine the quasienergy spectrum in a Floquet system, we computed the eigenvalue spectrum via diagonalization of the

corresponding Hamiltonian of the system (Fig. S4b). Beside the very good overall agreement, one can see some minor discrepancies. For instance, in the Fourier method based on propagation data some energy features are slightly blurred, which is a result of the spectral leakage caused by relying on finite propagation data. However, this effect could be reduced by using more propagation steps or employing an additional peak-extraction of the Fourier transformed data. Further, some of the theoretically expected energies have not been obtained due to a negligible overlap of the single-site excitation with some of the Floquet eigenmodes. However, for our system, most of the quasienergy features are successfully captured with the Fourier method.

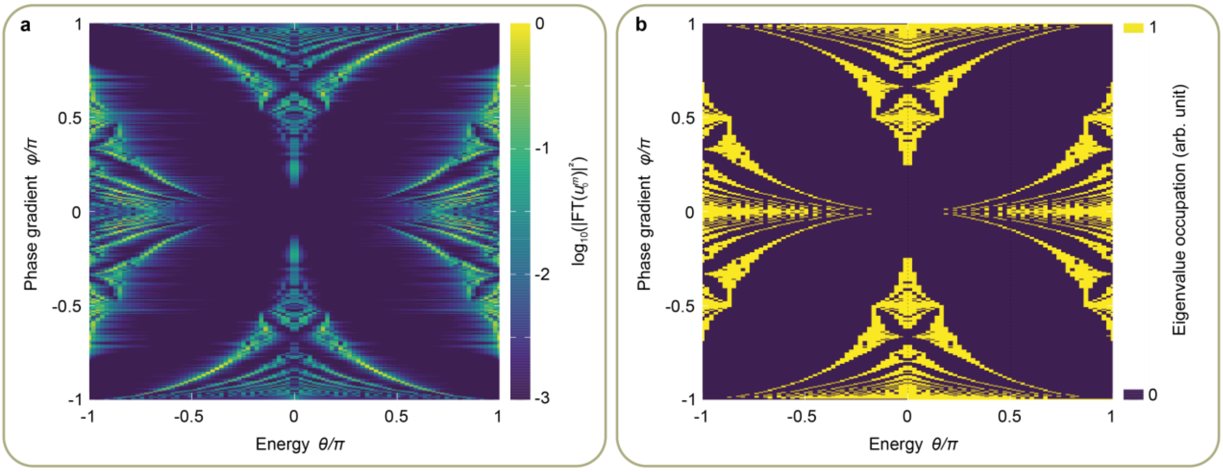

Fig. S4 | Eigenvalue extraction: Fourier method vs. diagonalization. a, Floquet Hofstadter butterfly from the Fourier method, based on the theoretical propagation data. **b**, Floquet Hofstadter butterfly from numerical diagonalization of the corresponding Hamiltonian. When using the diagonalization method, there are only two levels: 1, when there is a corresponding energy eigenvalue, and 0 otherwise.

4 Non-Hermitian delocalization transition

In this section, we derive the transition point for the topological triple phase transition. The topological triple phase transition is obtained for the non-Hermitian case of equations (2-3) in the SI, with $h \neq 0$ inducing a non-Hermitian anisotropy known from the non-Hermitian skin effect[4,8,9]. The gain/loss modulation $\lambda^{\pm 1}$ is analogous to the imaginary gauge field in the Hatano-Nelson model[10]. In fact, after the imaginary (non-unitary) gauge transformation

$$u_n \to u_n \exp(-hn), \qquad v_n \to \exp(-hn)\, v_n, \tag{16}$$

the non-Hermitian Floquet dynamics reduces to the Hermitian case with $h = 0$. Hence, one can observe the non-Hermiticity induced delocalization (metal-insulator) transition like in the Hatano-Nelson model. We consider a value $\beta > \pi/4$. Then for $h = 0$, the quasienergy spectrum is entirely real and all modes are localized with the inverse localization length $1/\gamma$ given by equation (10) of the SI. Now consider the gauge h as a control parameter. As h is slightly increased, such that h is smaller than $1/\gamma$, the quasienergy spectrum remains entirely real; the effect of non-vanishing h is to induce a spatially asymmetric localization of the modes. As h approaches the critical value $h_c = 1/\gamma$, the critical point is attained. For $h > h_c$ under periodic boundary conditions all wave functions are extended, the quasienergy spectrum becomes complex and it is described by closed loops in complex energy plane (see e.g. Fig.4a in the main manuscript). As shown below, the quasienergies emerge in complex conjugate pairs, like in a PT symmetry breaking phase transition. From the view of topology, in the localized phase, the winding number must vanish[11], while it can be non-zero in the delocalized phase for a base energy point chosen inside one of the closed loops of the complex energy spectrum.

The spectral topological winding number w for a lattice with incommensurate disorder is defined as follows[11–14]. Let us consider a sequence of rational numbers $\varphi_l = p_l/q_l$, with $q_{l+1} \geq q_l$ and $p_{l+1} \geq p_l$ that approximates the irrational number φ, i.e. $\varphi = \lim_{l\to\infty} \varphi_l$. For example, when φ is the inverse of the golden mean like in our experiment, i.e. $\varphi = (\sqrt{5} - 1)/2$, the sequence φ_l is given in terms of Fibonacci numbers $p_l = 0,1,1,2,3,5,8,13,21,24,55, \ldots$ with $q_l = p_{l+1}$. For $\varphi = \varphi_l$, the two-step propagator U (see Eq.(19) in the SI) is spatially periodic with the period $L = 2q_l$ describing a lattice with a commensurate potential. For large enough l, let us consider a lattice comprising $L = 2q_l$ sites under periodic (ring) boundary conditions with an applied magnetic flux ϑ, i.e. $(u, v)_{n+L} = (u, v)_n \exp(i\vartheta)$. This is basically equivalent to considering a ring geometry without a magnetic flux but with added terms $\pm\vartheta/(2L)$ to the phases ϕ_u and ϕ_v,

as discussed in the main manuscript. Indicating by $H(\vartheta/L) = i \log U$ the corresponding effective matrix Hamiltonian, the winding w with respect to a point-gap quasienergy θ_B is defined as

$$w = \lim_{L\to\infty} \frac{1}{2\pi i} \int_0^{2\pi} \frac{\partial}{\partial\vartheta} \log\det\left[H\left(\frac{\vartheta}{L}, h\right) - \mathbb{I}\cdot\theta_{\mathrm{B}}\right] \mathrm{d}\vartheta.$$

Physically, the winding number is related to the flow (spectral rotations) of the eigenenergies of $H(\vartheta/L)$ in the complex plane with respect to θ_B as ϑ continuously varies from zero to 2π [9–12]. As in reference[11], in our analysis we used $\theta_B = 0$. In the localized phase, the quasienergy spectrum is insensitive to the applied magnetic flux ϑ and thus $w = 0$: in this case the quasienergy spectrum remains entirely real and the system does not show the non-Hermitian skin effect. Conversely, in the delocalized phase the quasienergy spectrum becomes complex and describes a set of closed loops in the complex plane (Fig. 4a in the main text), one of which encircling the base energy $\theta_B = 0$ and corresponding to a winding $w = 1$. In this case, the system displays the skin effect.

5 PT symmetry

In this section we show the PT symmetry of the non-Hermitian Floquet quasicrystal, which shows the topological triple phase transition. In our model, the PT symmetry is hidden and can be unravelled after a suitable basis rotation[15]. To this aim, let us introduce the compact notation

$$|\psi^{(m)}\rangle \equiv \sum_n \begin{pmatrix} u_n^{(m)} \\ v_n^{(m)} \end{pmatrix} |n\rangle, \tag{17}$$

for the lattice site amplitudes in the system at time step m in the spatial representation $|n\rangle$, where n denotes the lattice site number. The two-step map takes the form

$$|\psi^{(m+2)}\rangle = U|\psi^{(m)}\rangle, \tag{18}$$

where the 2x2 elements of the evolution operator U read

$$U_{11} = -\sum_n \sin^2(\beta)\exp(-2i\phi_n^u)\,|n\rangle\langle n| - \lambda^2\cos^2(\beta)\exp(i\pi\varphi/2)\,|n\rangle\langle n+2|, \tag{19a}$$

$$U_{12} = \sum_n i\sin(\beta)\cos(\beta)\exp(-2i\phi_n^u)\,|n\rangle\langle n| \\ + i\lambda^2\sin(\beta)\cos(\beta)\exp(i\pi\varphi/2)\,|n\rangle\langle n+2|, \tag{19b}$$

$$U_{21} = \sum_n i\sin(\beta)\cos(\beta)\exp(-2i\phi_n^v)\,|n\rangle\langle n| \\ + \frac{i}{\lambda^2}\sin(\beta)\cos(\beta)\exp(i\pi\varphi/2)\,|n\rangle\langle n-2|, \tag{19c}$$

$$U_{22} = -\sum_n \sin^2(\beta)\exp(-2i\phi_n^v)\,|n\rangle\langle n| \\ - \frac{1}{\lambda^2}\cos^2(\beta)\exp(i\pi\varphi/2)\,|n\rangle\langle n-2|. \tag{19d}$$

The system displays PT symmetry[16], provided that

$$PTU = U^{-1}PT, \tag{20}$$

for a unitary linear operator $P^2 = 1$ and an antiunitary operator $T^2 = 1$. To unravel the PT symmetry, we introduce the basis rotation

$$\begin{pmatrix} u_n^{(m)} \\ v_n^{(m)} \end{pmatrix} = R(\alpha)\begin{pmatrix} x_n^{(m)} \\ y_n^{(m)} \end{pmatrix}, \tag{21}$$

where $R(\alpha) = \exp(i\alpha\sigma_1)$ with first Pauli matrix σ_1 and the rotation angle α. Clearly, the basis rotation does not change the quasienergy spectrum of the Floquet system. Let $|\Psi^{(m)}\rangle$ denote the wavefunction in the rotated basis, i.e.

$$|\Psi^{(m)}\rangle \equiv \sum_n \begin{pmatrix} x_n^{(m)} \\ y_n^{(m)} \end{pmatrix} |n\rangle = R^{-1}(\alpha)|\psi^{(m)}\rangle. \tag{22}$$

In the rotated basis, the evolution equation reads

$$|\Psi^{(m+2)}\rangle = \mathcal{U}|\Psi^{(m)}\rangle, \tag{23}$$

with the evolution operator $\mathcal{U}$ in the rotated basis given by

$$\mathcal{U} = R^{-1}(\alpha)UR(\alpha) = R(-\alpha)UR(\alpha). \tag{24}$$

We choose the rotation angle for the basis as

$$\alpha = \frac{\pi}{4} - \frac{\beta}{2}. \tag{25}$$

Under such a choice of the rotation angle, one obtains

$$\begin{aligned}\mathcal{U}_{11} = -\sum_n \frac{\sin(\beta)}{2}\big((\sin(\beta)+1)\exp(-2i\phi_n^u) \\ + (\sin(\beta)-1)\exp(-2i\phi_n^v)\big)|n\rangle\langle n| \\ + \frac{1}{2}\lambda^2\cos^2(\beta)\exp(i\pi\varphi/2)\,|n\rangle\langle n+2| \\ + \frac{1}{2\lambda^2}\cos^2(\beta)\exp(i\pi\varphi/2)\,|n\rangle\langle n-2|,\end{aligned} \tag{26a}$$

$$\begin{aligned}\mathcal{U}_{12} = \sum_n \frac{i}{2}\sin(\beta)\cos(\beta)\,(\exp(-2i\phi_n^u) + \exp(-2i\phi_n^v))|n\rangle\langle n| \\ + \frac{i}{2}\lambda^2(\sin(\beta)+1)\cos(\beta)\exp(i\pi\varphi/2)\,|n\rangle\langle n+2| \\ + \frac{i}{2\lambda^2}(\sin(\beta)-1)\cos(\beta)\exp(i\pi\varphi/2)\,|n\rangle\langle n-2|,\end{aligned} \tag{26b}$$

$$\begin{aligned}\mathcal{U}_{21} = \sum_n \frac{i}{2}\sin(\beta)\cos(\beta)\,(\exp(-2i\phi_n^u) + \exp(-2i\phi_n^v))|n\rangle\langle n| \\ + \frac{i}{2}\lambda^2(\sin(\beta)-1)\cos(\beta)\exp(i\pi\varphi/2)\,|n\rangle\langle n+2| \\ + \frac{i}{2\lambda^2}(\sin(\beta)+1)\cos(\beta)\exp(i\pi\varphi/2)\,|n\rangle\langle n-2|,\end{aligned} \tag{26c}$$

$$\begin{aligned}\mathcal{U}_{22} = -\sum_n \frac{\sin(\beta)}{2}\big((\sin(\beta)+1)\exp(-2i\phi_n^v) \\ + (\sin(\beta)-1)\exp(-2i\phi_n^u)\big)|n\rangle\langle n| \\ + \frac{1}{2}\lambda^2\cos^2(\beta)\exp(i\pi\varphi/2)\,|n\rangle\langle n+2| \\ + \frac{1}{2\lambda^2}\cos^2(\beta)\exp(i\pi\varphi/2)\,|n\rangle\langle n-2|.\end{aligned} \tag{26d}$$

A direct computation of the inverse operator $\mathcal{U}^{-1}$ shows that the elements of $\mathcal{U}^{-1}$ are just the complex conjugates of those of $\mathcal{U}$. Therefore, one can find a PT symmetry operator that does not even involve any spatial reflection but only acts on the internal degrees of the state vector. For example, by introducing the parity and time-reversal operators as

$$P = \sigma_i, \qquad T = \sigma_i \mathcal{K}, \tag{27}$$

where σ_i is any of the Pauli matrices and $\mathcal{K}$ denotes complex conjugation, one has

$$PT = \mathcal{K}, \tag{28}$$

and thus

$$PT\mathcal{U} = \mathcal{U}^{-1} PT. \tag{29}$$

In the rotated basis the evolution operator is PT symmetric (albeit it does not show separately P and T symmetry) and its quasienergies thus appear in complex conjugate pairs, which we could confirm by numerical diagonalization of the evolution operator. In the original basis, this means that if $(u_n, v_n)^\top$ is a Floquet eigenstate of U with quasienergy θ, then

$$\begin{pmatrix} \tilde{u}_n \\ \tilde{v}_n \end{pmatrix} = R(\alpha)\mathcal{K}R(-\alpha)\begin{pmatrix} u_n \\ v_n \end{pmatrix} = R(2\alpha)\begin{pmatrix} \tilde{u}_n^* \\ \tilde{v}_n^* \end{pmatrix} = \begin{pmatrix} \cos\beta & i\sin\beta \\ i\sin\beta & \cos\beta \end{pmatrix}\begin{pmatrix} \tilde{u}_n^* \\ \tilde{v}_n^* \end{pmatrix}, \tag{30}$$

is a Floquet eigenstate with quasienergy θ^*. This general result holds regardless of the values of λ and φ, i.e., even for a non-unitary quantum walk in an incommensurate potential ($\lambda \neq 1$ and φ irrational). In a periodic lattice (φ rational), where the wave functions are extended (Bloch states), owing to the skin effect under periodic boundary conditions the PT symmetry is broken for any infinitesimal change of λ from 1. However, for an incommensurate potential (φ irrational Diophantine) the skin effect is counteracted by the exponential localization of the wave function, until the localization-delocalization transition arises at the critical value $h_c(\beta)$ of the imaginary gauge field.

References

1. Regensburger, A. *et al.* Parity-time synthetic photonic lattices. *Nature* **488**, 167–171 (2012).
2. Schreiber, A. *et al.* Photons walking the line: a quantum walk with adjustable coin operations. *Phys. Rev. Lett.* **104**, 050502 (2010).
3. Wimmer, M., Price, H. M., Carusotto, I. & Peschel, U. Experimental measurement of the Berry curvature from anomalous transport. *Nat. Phys.* **13**, 545–550 (2017).
4. Weidemann, S. *et al.* Topological funneling of light. *Science.* **368**, 311–314 (2020).
5. Knight, P. L., Roldán, E. & Sipe, J. E. Quantum walk on the line as an interference phenomenon. *Phys. Rev. A* **68**, 020301 (2003).
6. Jeong, H., Paternostro, M. & Kim, M. S. Simulation of quantum random walks using the interference of a classical field. *Phys. Rev. A* **69**, 012310 (2004).
7. Serge Aubry, G. A. Analyticity breaking and Anderson localization in incommensurate lattices. *Ann. Isr. Phys. Soc* **3**, 18 (1980).
8. Yao, S. & Wang, Z. Edge States and Topological Invariants of Non-Hermitian Systems. *Phys. Rev. Lett.* **121**, 086803 (2018).
9. Helbig, T. *et al.* Generalized bulk–boundary correspondence in non-Hermitian topolectrical circuits. *Nat. Phys.* **16**, 747–750 (2020).
10. Hatano, N. & Nelson, D.R. Localization Transitions in Non-Hermitian Quantum Mechanics. *Phys. Rev. Lett.* **77**, 570–573 (1996).
11. Gong, Z. *et al.* Topological Phases of Non-Hermitian Systems. *Phys. Rev. X* **8**, 031079 (2018).
12. Zeng, Q.-B. & Xu, Y. Winding numbers and generalized mobility edges in non-Hermitian systems. *Phys. Rev. Res.* **2**, 033052 (2020).
13. Cai, X. Boundary-dependent self-dualities, winding numbers, and asymmetrical localization in non-Hermitian aperiodic one-dimensional models. *Phys. Rev. B* **103**, 1–12 (2021).
14. Longhi, S. Non-Hermitian Maryland model. *Phys. Rev. B* **103**, 224206 (2021).
15. Longhi, S. Non-Bloch PT symmetry breaking in non-Hermitian photonic quantum walks. *Opt. Lett.* **44**, 5804 (2019).
16. Bender, C. M. & Boettcher, S. Real spectra in non-hermitian hamiltonians having PT symmetry. *Phys. Rev. Lett.* **80**, 5243–5246 (1998).
